# Supplementary material for: Zinc tolerance through glutathione import strikes a fine balance between protection and damage in Streptococcus mutans
Source: Microbiology (Reading). 2026 Aug 10;172(8):001754. doi: 10.1099/mic.0.001754 (PMC13456638; doi:10.1099/mic.0.001754)
Supplement: Supplementary file 1 [file mic-172-01754-s001.pdf]

Supplemental Material

TABLE S1. Primers used in this study

Primer	Sequence (5' – 3')	Application
**1942c.del.3arm.rev**	AGGTTCATCAACGCCTTTGGC	*gshT* deletion
**1942c.del.3arm.fwd**	CCTTGGAGGATCCAGCTATCTTCCAG	*gshT* deletion
**1942c.5arm.rev.del**	GATTTGTTGTGGATCCAACAGTCGCAA	*gshT* deletion
**1942c.5arm.fwd.del**	GCGATCATCGTGGTGCTTTAGTAG	*gshT* deletion
**1942c.3arm.screen**	GTCATGGTCATCACACCAACAGTTAAC	*gshT* deletion screening
**1942c.5arm.screen**	CCCATCCTCTTTCTTTATGACGACAAG	*gshT* deletion screening
**zccE.checkF**	GTATTATCAACAACGTATTGCA	*zccE* deletion screening
**zccE.checkR**	TTATTGCTTACGAAGATATCG	*zccE* deletion screening
**gshTcomp.GA.erm.fwd**	ATATTTTACTGGATGAATTGTTTTAGTAGACTTAACAATG ATATTCGTGATAG	Creation of $gshT^{comp}$
**gshTcomp.GA.erm.rev**	GCCATTTATTATTTCCTTCCTCTTTTACTCAGATTAGACT AGGTGAC	Creation of $gshT^{comp}$
**gshT.qrt.fwd**	GTTCCAACAGTCGCAAGGGT	qRT-PCR of *gshT*
**gshT.qrt.rev**	GAGCGGTTGCTTTTGCCTCA	qRT-PCR of *gshT*
**tcyA.qrt.fwd**	ATTCAGTTGGCGGGCAGTCT	qRT-PCR of *tcyA*
**tcyA.qrt.rev**	CAAGATCGGCGTCCCCCTTA	qRT-PCR of *tcyA*
**gyrA.qrt.fwd**	CTCCTGACAAGCCGCATAAA	qRT-PCR of *gyrA*
**gyrA.qrt.rev**	GCTCATACGCGCTTCTGTATAA	qRT-PCR of *gyrA*

Table S2. R packages used in data analysis and visualization

R package	Citation
tidyverse	Wickham et al. 2019 (1)
ggtext	Wilke & Wiernik, 2022 (2)
rstatix	Kassambara, 2023 (3)
dbplyr	Wickham et al., 2026 (4)
ggpubr	Kassambara, 2023 (5)
ggsignif	Ahlmann-Eltze & Patil, 2021 (6)
multcomp	Hothorn et al., 2008 (7)
magrittr	Bache & Wickham, 2022 (8)
emmeans	Lenth et al., 2025 (9)
lme4	Bates et al., 2015 (10)

Fig S1

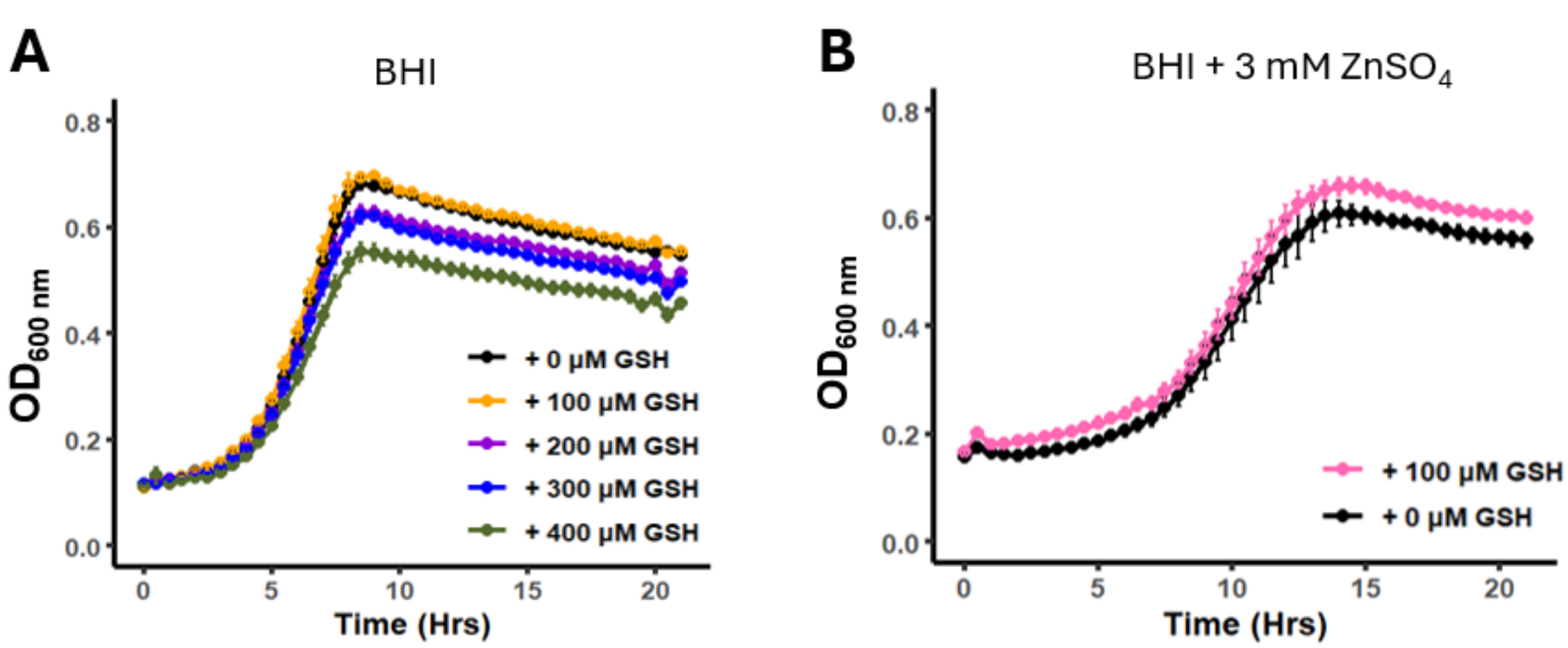

Fig. S1. Glutathione supplementation minimally improves *S. mutans* UA159 growth on a sub-inhibitory concentration of zinc. (A) Growth of UA159 in BHI supplemented with increasing concentrations of glutathione (GSH). (B) Growth of UA159 in BHI + 3 mM $ZnSO_4$ with or without 100 µM glutathione (GSH) supplementation. Error bars are represented by SEM where n≥8.

Fig S2

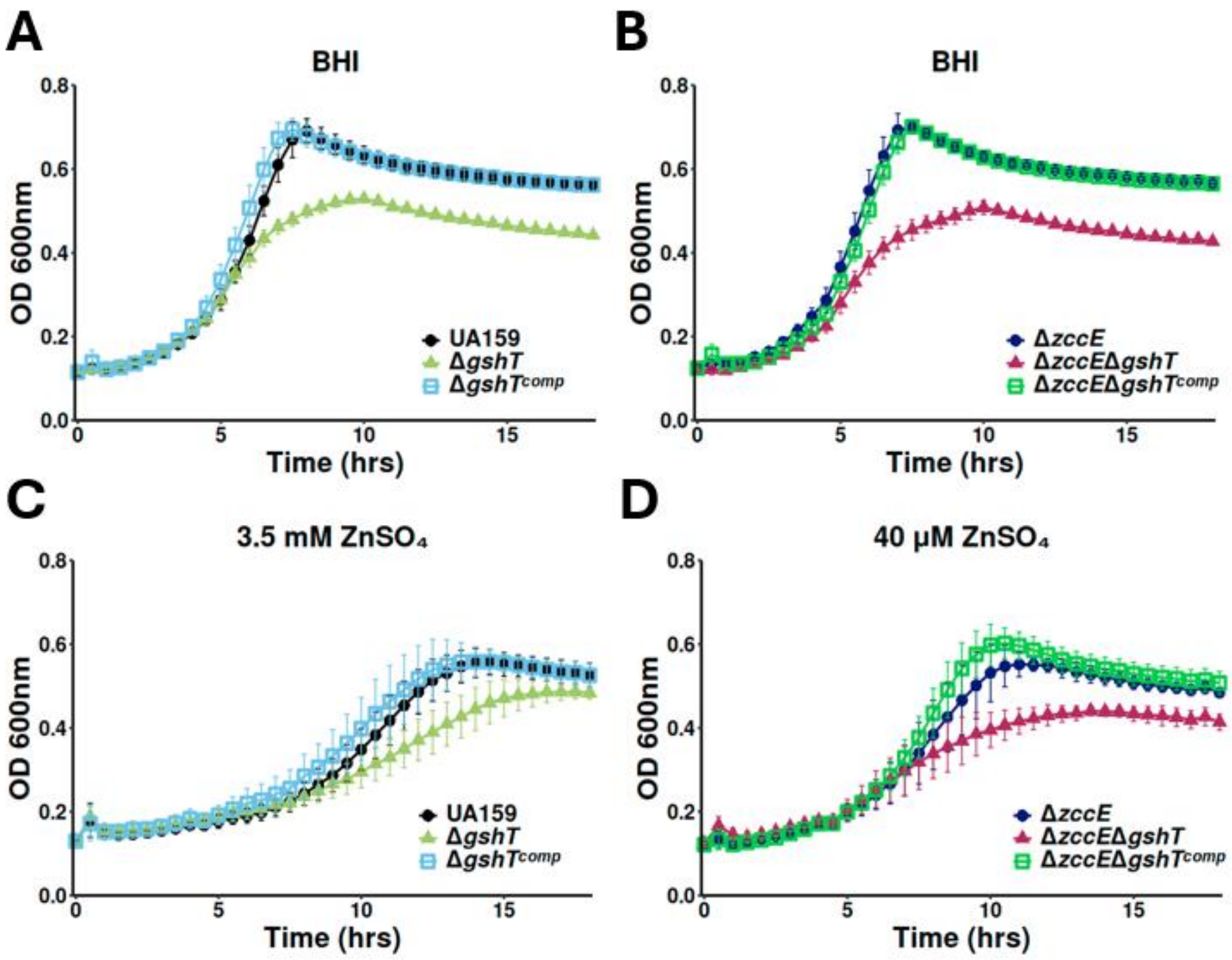

Fig. S2. Growth of *S. mutans* UA159, Δ*gshT*, Δ*gshT*^{comp}, Δ*zccE*, Δ*zccE*Δ*gshT* and Δ*zccE*Δ*gshT*^{comp} in plain BHI (A-B) or BHI + 3.5 mM $ZnSO_4$ (C) or BHI + 40 µM $ZnSO_4$ (D). Error bars are represented by SEM where n≥8.

References

1. Wickham H, Averick M, Bryan J, Chang W, McGowan LD, François R, et al. Welcome to the Tidyverse. Journal of Open Source Software. 2019 Nov 21;4(43):1686. doi:10.21105/joss.01686

2. Wilke CO, Wiernik BM. ggtext: Improved Text Rendering Support for "ggplot2" [Internet]. 2020 [cited 2026 May 18]. p. 0.1.2. Available from: https://CRAN.R-project.org/package=ggtext doi:10.32614/CRAN.package.ggtext

3. Kassambara A. rstatix: Pipe-Friendly Framework for Basic Statistical Tests [Internet]. 2025 [cited 2026 May 18]. Available from: https://cran.r-project.org/web/packages/rstatix/index.html

4. Wickham H, Girlich M, Ruiz E, Software P, PBC. dbplyr: A "dplyr" Back End for Databases [Internet]. 2026 [cited 2026 May 18]. Available from: https://cran.r-project.org/web/packages/dbplyr/index.html

5. Kassambara A, Business LE (Faculty of E and, Debrecen U of, Hungary). ggpubr: "ggplot2" Based Publication Ready Plots [Internet]. 2026 [cited 2026 May 18]. Available from: https://cran.r-project.org/web/packages/ggpubr/index.html

6. Ahlmann-Eltze C, Patil I. ggsignif: R Package for Displaying Significance Brackets for 'ggplot2 [Internet]. 2021 [cited 2026 May 18]. Available from: https://doi.org/10.31234/osf.io/7awm6 doi:10.31234/osf.io/7awm6

7. Hothorn T, Bretz F, Westfall P. Simultaneous inference in general parametric models. Biom J. 2008 Jun;50(3):346–63. doi:10.1002/bimj.200810425 PubMed PMID: 18481363.

8. Bache SM, Wickham H, Henry L, Software P. magrittr: A Forward-Pipe Operator for R [Internet]. 2026 [cited 2026 May 18]. Available from: https://cran.r-project.org/web/packages/magrittr/index.html

9. Lenth RV, Piaskowski J, Banfai B, Bolker B, Buerkner P, Giné-Vázquez I, et al. emmeans: Estimated Marginal Means, aka Least-Squares Means [Internet]. 2026 [cited 2026 May 18]. Available from: https://cran.r-project.org/web/packages/emmeans/index.html

10. Bates D, Mächler M, Bolker B, Walker S. Fitting Linear Mixed-Effects Models Using lme4. Journal of Statistical Software. 2015 Oct 7;67:1–48. doi:10.18637/jss.v067.i01
